# Ecologic Niche Modeling and Spatial Patterns of Disease Transmission

**DOI:** 10.3201/eid1212.060373

**Published:** 2006-12

**Authors:** A. Townsend Peterson

**Affiliations:** * University of Kansas, Lawrence, Kansas, USA

**Keywords:** Ecologic niche modeling, geographic distribution, spatial pattern, perspective

## Abstract

Ecologic niche modeling (ENM) is a growing field with many potential applications to questions regarding the geography and ecology of disease transmission. Specifically, ENM has the potential to inform investigations concerned with the geography, or potential geography, of vectors, hosts, pathogens, or human cases, and it can achieve fine spatial resolution without the loss of information inherent in many other techniques. Potential applications and current frontiers and challenges are reviewed.

## Abstract 2

TOC Summary: This technique can be used to study the geography and ecology of disease transmission.

The emerging and evolving field of landscape epidemiology has explored techniques for summarizing spatial patterns in disease transmission data. These techniques seek spatial patterns at some level of generalization or averaging and then summarize overall patterns and trends in the form of a smoothed surface. Techniques typically applied to these challenges include splining and kriging, as well as smoothing based on average values within coarser-grained windows across landscapes (*1**–**3*). These approaches always involve some loss of resolution to smooth the surfaces, and some degree of averaging is involved (Figure).

**Figure:**
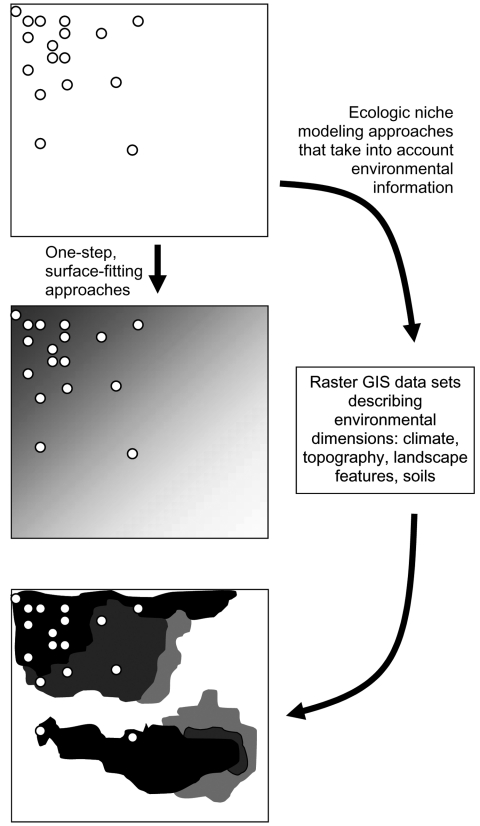
Hypothetical example of a species’ known occurrences (circles) and inferences from that information. The middle panel shows the pattern that would result from a surface-fitting or smoothing algorithm, and the bottom panel shows the ability of ecologic niche modeling approaches to detect unknown patterns in biologic phenomena based on the relationship between known occurrences and spatial patterns in environmental parameters. GIS, geographic information system.

Although these approaches provide simple summaries of spatial patterns, they do not often succeed in illustrating true levels of complexity and heterogeneity that characterize biologic landscapes. Disease transmission cycles are composite phenomena that represent interactions between sets of species: hosts, vectors, and pathogens. The complexities of spatial occurrence of disease will represent the combination of complexities of occurrence of the component species, as well as effects of chance events. Thus, broad-trend generalizations such as those produced using the smoothing techniques mentioned above are unlikely to lead to novel insights and new understanding of complex systems. The approach advocated in this report improves the pattern summary by estimating species-specific ecologic niches. In this way, the complex influences of environmental variation on species' distributions and their translation into disease transmission patterns can be appreciated in greater detail (Figure).

## Ecologic Niche Modeling (ENM)

Joseph Grinnell originated the concept of ecologic niches and was the first to explore the connections between ecologic niches and geographic distributions of species (*4*). His idea, translated into more modern terminology, was that the ecologic niche of a species is the set of conditions under which the species can maintain populations without immigration of individuals from other areas. A more complete discussion of the concept of ecologic niches and their mapping onto the geographic distributions of species has been provided elsewhere (*5*).

Use of the ENM approach has grown considerably in the biodiversity community in recent years (*6**–**10*). The idea is that known occurrences of species across landscapes can be related to raster geographic information system coverages summarizing environmental variation across those landscapes to develop a quantitative picture of the ecologic distribution of the species. ENM characterizes the distribution of the species in a space defined by environmental parameters, which are precisely those that govern the species' geographic distribution under Grinnell's definition.

A particular strength of ENM is its independence from any particular landscape. ENM can be used to identify potential distributional areas on any landscape: unsampled or unstudied portions of the native landscape, areas of actual or potential invasion by a species with an expanding range, or changing potential distributional areas as a consequence of change (e.g., land use change or climate change). Thus, ENM represents a powerful tool for characterizing ecologic and geographic distributions of species across real-world landscapes.

## Applications to Disease Systems

In recent years, the ENM approach has seen several prototype applications to disease transmission systems by public health and epidemiology specialists who have been willing to explore novel ideas and approaches. I outline what the technique has to offer to the field and provide citations of example publications for each benefit and use.

### Understanding Ecology of Diseases

In many cases, the details of ecologic parameters associated with occurrences of diseases or of species participating in disease transmission (e.g., vectors, hosts, pathogens) may be unclear because of small sample sizes, biased reporting, or simply lack of detailed geographic or ecologic analysis. ENM encompasses a suite of tools that relate known occurrences of these species or phenomena to raster geographic information system layers that summarize variation in several environmental dimensions. The result is an objective, quantitative picture of how what is known about a species or phenomenon relates to environmental variation across a landscape. Studies using these approaches include an examination of ecologic differences among different Chagas disease vectors in Brazil (*11*) and a characterization of ecologic features of outbreaks of hemorrhagic fever caused by Ebola and Marburg viruses (*12**,**13*).

### Characterizing Distributional Areas

A next step in applying ENM approaches to understanding disease systems is characterizing geographic distributions. Here, ENM (or something akin to it) is used to investigate landscapes for areas that meet the ecologic requirements of the species. The result is an interpolation between known sampling locations informed by observed associations between the species and environmental characteristics. Previous attempts to characterize geographic distributions of species in the disease realm have demonstrated the potential of the approach but have not always used the most powerful inferential techniques available (*14**,**15*). In at least 1 case (*14*), the methods used failed to generalize and predict into areas of sparse sampling. ENM produces statistically robust predictions of geographic distributions of species or phenomena (even in unsampled areas), greatly exceeding expectations under random (null) models. Numerous examples of applications of this functionality to disease systems have been published (*11**–**13**,**16**–**22*).

### Identifying Areas of Potential Invasion in Other Regions

ENMs characterize general environmental regimes under which species or phenomena may occur. To the extent that the model is appropriately and correctly calibrated, it may be used to seek areas of potential distribution. Thus, ENMs can be used to identify areas that fit the ecologic bill for a species, even if the species is not present there. This approach has seen extensive experimentation and testing in the biodiversity realm (*8**,**23*), but applications to disease transmission have as yet been few. One study attempted to identify the particular species in the Anopheles gambiae complex that was responsible for the large-scale South American malaria outbreaks in the early 20th century (*19*), and another evaluated the geographic potential of a possible monkeypox host (Cricetomys spp.) in North America (*24*).

### Anticipating Risk Areas with Changing Climates

A logical extension of using ENMs to identify potential distributional areas is to address the question of likely geographic shifts in distributional areas of species or phenomena under scenarios of climate change or changing land use (*25*). This approach has seen considerable attention in the biodiversity realm, with both tests and validations (*26**–**28*), and with broad applications across faunas and floras (*29**–**32*). In the disease world, applications have been few, although 1 study used likely climate change–mediated range shifts to hypothesize the identity of Lutzomyia vectors of recent leishmaniasis outbreaks in southern Brazil (*21*).

### Identifying Unknown Vectors or Hosts

ENM approaches can be applied to various parts of disease transmission cycles (e.g., overall case distribution, reservoir host distribution, vector distribution) to identify unknown elements in systems. The geography of overall case distributions can provide an indication of which clades are potential reservoirs and which are not. A first application was an attempt to identify mammalian hosts of the Triatoma protracta group of Chagas disease vectors in Mexico (*22*), which succeeded in anticipating the mammal hosts of 5 of 5 species for which a test was possible. Further exploration of this possible application of ENM methods has focused on the mysterious long-term reservoir of the filoviruses (Ebola and Marburg viruses) by comparing African mammal distributions with those of filovirus-caused disease outbreaks (*33*).

## Discussion

### Current Challenges in ENM

ENM, although it has old roots (*4*), is nonetheless a relatively new tool in distributional ecology and biogeography. Only a few recent studies have compared the performance of different methodologic approaches under the ENM rubric (*34**–**37*). As such, numerous challenges remain in terms of refining approaches toward a more powerful and synthetic methodology.

One central challenge is that of choosing modeling methods appropriate to a particular question, in the sense of discerning interpolation challenges from extrapolation challenges. In a recent comparative study focused on interpolation, which inferred details of patterns of presence and absence on a densely sampled landscape, several techniques that have internal controls on overfitting were superior (*34*). Extrapolative challenges, such as predicting potential distribution of invasive species, anticipating species' responses to global climate change, and identifying unknown reservoirs or vectors, require different qualities of modeling algorithms; different methods therefore appear to emerge as superior, according to the particular challenge (*5*). This balance of ability to interpolate accurately versus ability to extrapolate effectively remains a challenge for the ENM methods.

A second frontier that includes yet-to-be-resolved details for ENM is that of testing and evaluating model results. Currently accepted approaches center on the ability to predict independent test occurrence data in the smallest area predicted (*34**,**38*). However, efficient predictions can be poor descriptors of a species' geographic range. Simpler techniques that place greater emphasis on minimizing the omission of known occurrences may be more appropriate. Pairing significance tests (which demonstrate that the coincidence between a prediction and test data is better than that achieved by random or null models) with setting minimum performance criteria (which ensure that that the prediction is accurate enough to meet the needs of the study) is probably the best approach (*38*). However, these methods have yet to be agreed upon broadly in the ENM community.

### Current Challenges in Applications of ENM to Disease Systems

Beyond methodologic challenges, several issues remain to be addressed for full application of ENM methods to disease systems. The first, and perhaps most important, is understanding the role of scale in space and time. Preliminary explorations suggest that proper matching of temporal and spatial scales in analyses may offer particular opportunities for precise and accurate prediction of the behavior of disease phenomena (*39*). Similarly, proper choice of environmental datasets requires further exploration. Climate data provide longer temporal applicability, but remotely sensed data that summarize aspects of surface reflectance can provide finer spatial resolution, and may measure aspects of ecologic landscapes that climate parameters alone may not capture (*40*). Such issues will be resolved only through further exploration and testing with predictive challenges for diverse disease systems.

Finally, because disease transmission systems often represent complex interactions among multiple species (e.g., vectors, hosts, pathogens), options exist for how they should be analyzed and modeled. Simple focus on disease occurrences, such as human cases, treats the entire transmission system as a black box and as such gives an overall picture of the ecology of the transmission chain of that disease (*12*). An alternative, however, is modeling each component species in the transmission system and then assembling the component ENMs into a geographic picture of the transmission system (*22*). Each of these approaches has its relative advantages and disadvantages, but a best-practices method has yet to be established, pending further testing and exploration.

## Conclusions

The emerging field of ENM applied to questions of ecologic and geographic characteristics of disease systems has considerable potential. In particular, it can solve several problems of spatial resolution of summaries of geographic risk for disease. In sharp contrast to surface-fitting approaches to the same questions, ENM does not lose resolution to generalize and produce a result. Rather, ENM can achieve fine-scale resolution of distributions limited only by the spatial precision of the input occurrence data and the input environmental datasets. This characteristic makes possible a clear improvement in the spatial resolution that is possible in representing spatial patterns in disease risk.

ENM is in the early stages of being explored for its potential for illuminating unknown phenomena in the world of disease transmission. The extensive explorations of ENM in the biodiversity field, however, serve as a benchmark of quality and acceptance for the technique. It can, once tested and prototyped extensively in the disease realm, offer a much-improved representation of spatial patterns in distributions of species or other phenomena.
